# High frequency of PDGFRA and MUC family gene mutations in diffuse hemispheric glioma, H3 G34-mutant: a glimmer of hope?

**DOI:** 10.1186/s12967-022-03258-1

**Published:** 2022-02-02

**Authors:** Wanming Hu, Hao Duan, Sheng Zhong, Jing Zeng, Yonggao Mou

**Affiliations:** 1 Department of Pathology, State Key Laboratory of Oncology in South China, Collaborative Innovation Center for Cancer Medicine, Sun Yat-Sen University Cancer Center, Guangzhou, China; 2 Department of Neurosurgery, State Key Laboratory of Oncology in South China, Collaborative Innovation Center for Cancer Medicine, Sun Yat-Sen University Cancer Center, Guangzhou, China

**Keywords:** Diffuse hemispheric glioma, H3G34R/V, H3K27M, PDGFRA, MUC, Immune infiltration, Survival

## Abstract

**Background:**

Diffuse hemispheric glioma H3 G34-mutant (G34-DHG) is a new type of pediatric-type diffuse high-grade glioma in the fifth edition of the WHO Classification of Tumors of the Central Nervous System. The current treatment for G34-DHG involves a combination of surgery and conventional radiotherapy or chemotherapy; however, the therapeutic efficacy of this approach is not satisfactory. In recent years, molecular targeted therapy and immunotherapy have achieved significant benefits in a variety of tumors. In-depth understanding of molecular changes and immune infiltration in G34-DHGs will help to establish personalized tumor treatment strategies. Here, we report the clinicopathological, molecular and immune infiltration characteristics of G34-DHG cases from our center along with cases from the HERBY Trial and the Chinese Glioma Genome Atlas database (CGGA).

**Methods:**

Hematoxylin–eosin (HE) and immunohistochemistry (IHC) staining were used to present the clinicopathological characteristics of 10 Chinese G34-DHG patients treated at our institution. To address the molecular characteristics of G34-DHG, we performed whole-exome sequencing (WES) and RNA sequencing (RNA-seq) analyses of 5 patients from our center and 3 Chinese patients from the Chinese Glioma Genome Atlas (CGGA) database. Additionally, 7 European G34-DHG patients from the HERBY Trail were also subjected to analyses, with 7 cases of WES data and 2 cases of RNA-seq data. Six G34-DHG patients from another organization were used as external validation.

**Results:**

WES showed a high frequency of *PDGFRA* mutation in G34-DHGs (12/15). We further identified frequent mutations in *MUC* family genes in G34-DHGs, including *MUC16* (8/15) and *MUC17* (8/15). Although no statistical difference was found, *PDGFRA* mutation tended to be an indicator for worse prognosis whereas *MUC16*/*MUC17* mutation indicated a favorable prognosis in G34-DHGs. RNA sequencing results revealed that most G34-DHG are considered to be immune cold tumors. However, one patient in our cohort with *MUC16* mutation showed significant immune infiltration, and the total overall survival of this patient reached 75 months.

**Conclusions:**

Our results demonstrate that G34-DHG is a new high-grade glioma with high frequency of *PDGFRA* and *MUC* gene family mutations. PDGFRA may serve as an indicator of poor prognosis and an effective therapeutic target. Moreover, *MUC16* tends to be a favorable prognostic factor and indicates high immune infiltration in certain patients, and these findings may provide a new direction for targeted therapy and immunotherapy of patients with G34-DHGs.

**Supplementary Information:**

The online version contains supplementary material available at 10.1186/s12967-022-03258-1.

## Background

Pediatric-type diffuse high-grade glioma (pHGG) is a highly aggressive brain tumor with a poor prognosis that accounts for approximately 8–15% of all central nervous system tumors in children and adolescents [1]. The widespread use of high-throughput sequencing, genetic profiling and epigenetic analysis has greatly increased our understanding of the cell origin, pathogenesis and biological characteristics of pHGG. Two types of heterozygous somatic mutations in *H3F3A*, which encodes histone H3, were firstly identified in pHGG in 2012 [2]. These mutations lead to an amino acid substitution at key residues (K27M or G34R/G34V). Subsequent studies found that K27M mutation presented in both H3F3A and HIST1H3B/C genes, but G34R/V-mutant gliomas were characterized by recurrent glycine-to-arginine/valine alterations at codon 34 (G34R/V) only in the H3F3A gene. The fifth edition of the WHO Classification of Tumors of Central Nervous System in 2021 defined the standard name of G34R/V-mutant gliomas as diffuse hemispheric glioma H3 G34-mutant, classified as WHO grade 4.

Most diffuse hemispheric glioma H3 G34-mutant tumors (G34-DHGs) carry *TP53* mutations and *ATRX* deletions [2], with hypermethylation at the OLIG2 locus, leading to the absence of OLIG2 expression [3]. The current treatment for these tumors is surgery, radiotherapy and chemotherapy. However, the efficacy of this therapeutic strategy is not satisfactory. In-depth understanding of the molecular changes and immune infiltration in G34-DHGs will help to establish personalized tumor treatment strategies.

In this study, we conducted a comprehensive analysis of G34-DHGs at the clinicopathological and genetic level. Our research uncovered new molecular characteristics of this unique tumor, which provide potential opportunities to develop individualized therapy.

## Methods

### Patient cohort

The institutional ethical committee of Sun Yat-sen University Cancer Center (SYSUCC) approved this study, and written informed consent was obtained from all patients. A flow diagram of patient selection is shown in Additional file 1: Figure S1.

Ten H3F3A G34-mutated patients were selected from the clinicopathology database on the basis of *H3F3A* Sanger sequencing results, including nine patients with G34R mutation and one patient with G34V mutation. Pathological specimens of all patients were independently reviewed by two senior neuropathologists according to the criteria of the 2021 WHO Classification of Tumors of Central Nervous System [4]. All patients met the criteria for diffuse hemispheric glioma, H3 G34-mutant. Clinicopathological characteristics of patients including age, sex, chief complaint, site of lesion, treatment, IDH/TERT/MGMT promoter methylation status, and survival status were retrospectively reviewed.

Five cases had sufficient fresh tumor samples for whole-exome sequencing (WES) and RNA sequencing (RNA-seq); freshly frozen tumor tissues were subjected to WES and RNA-seq analyses, and corresponding blood samples were subjected to WES analyses. We also obtained WES and RNA-seq data of three Chinese patients from the Chinese Glioma Genome Atlas (CGGA) database and seven European patients from the HERBY Trial (study BO25041; clinicaltrials.gov NCT01390948).

For survival analysis, we examined overall survival (OS) of the total 20 patients with G34-DHG, along with 21 cases with *IDH*-mutant high-grade glioma and 34 cases with H3K27M-mutant diffuse midline glioma at our institution.

### Immunohistochemistry

Immunohistochemical staining was performed using an immunostaining system (BenchMark ULTRA system, Ventana-Roche, Switzerland) with primary antibodies against GFAP (1:200, ZSGB-BIO, Beijing, China), OLIG2 (1:100, ZSGB-BIO), ATRX (1:200, ZSGB-BIO), IDH1 R132H (1:100, MX031, Fuzhou, China), H3K27M (1:1000, Millipore, Temecula, CA, USA), p53 (1:1000, Dako, Glostrup, Denmark) and Ki67 (1:200; Dako). Appropriate positive controls were included.

### Methylation-specific PCR analysis

Methylation-specific PCR was conducted using an EZ DNA Methylation Kit (Zymo Research, Irvine, CA, USA) to determine the methylation status of the MGMT promoter, as described previously [5].

### WES

DNA was extracted from fresh tissues, paraffin-embedded tissue and peripheral blood. WES was performed using a targeted capture approach with the Agilent SureSelect Human All Exon Kit (Santa Clara, CA, USA) followed by massively parallel sequencing of enriched fragments on the Illumina HiSeq2500 by Gene Denovo Biotechnology Co. Tumor and corresponding blood leukocyte DNA samples had an average sequencing depth of the target exonic region of > 200×. For a big panel targeting 539 and a small panel targeting 135 tumor genes, the sequencing depth was 2000×.

### Somatic variant identification

All the sequence reads were mapped according to the human reference genome GRCh37 using BWA-MEM with default parameters. Following the GATK standard protocol, realignment and recalibration were performed for BAM files after removing PCR duplicates. Single-nucleotide variations (SNVs) were identified with MuTect in the GATK suite. Using white blood cell data as controls, tumor sample data were then input into GATK to call somatic mutations (SNV). Finally, maftools was employed to visualize the mutational landscape of the results.

### Immune infiltration score calculation

To de-convolute immune components of tumor samples, the TIMER online tool (http://cistrome.org/TIMER/) was employed to evaluate the immune cell infiltration score using default settings. The immune infiltration score was later visualized and plotted by the R studio function pheatmap.

### Statistical analysis

Statistical analysis was performed using IBM-SPSS Statistics version 18.0 (IBM, NY, USA). Kaplan–Meier survival curves were generated to estimate OS. Survival differences were analyzed by the log-rank test.

## Results

### Clinical characteristics of G34-DHGs

The clinical characteristics of the 10 patients with G34-DHGs from SYSUCC are summarized in Table 1. The patient group included four male patients and six female patients. The patient age at initial diagnosis ranged from 13 to 25 years, and the median patient age was 20.5 years. The symptoms were the classic clinical features of brain tumors, including dizziness, headache, nausea, vomiting, weakness of right limbs and generalized tonic–clonic seizures dependent on the site of the lesion. All lesions were primarily located in cerebral hemispheres; the lesions were in the right hemisphere in five patients, the left hemisphere in four patients and bilateral hemisphere in one patient. Tumor invasion in the frontal lobe was detected in eight patients, and invasion in the parietal lobe was detected in three patients; temporal lobe involvement was observed in two patients and insular lobe involvement was observed in two patients. In addition, corona radiata, basal ganglia or corpus callosum were infiltrated by tumors in four patients.

**Table 1:** Clinical characteristics of 10 SYSUCC patients with H3 G34-mutant diffuse hemispheric gliomas

	Patient 1	Patient 2	Patient 3	Patient 4	Patient 5	**Patient 6**	**Patient 7**	**Patient 8**	**Patient 9**	**Patient 10**
Sex/age (years)	F/14	F/18	M/16	F/20	M/25	F/23	F/23	M/13	F/21	M/21
Chef complaint	Headache, nausea and vomiting	Headache, nausea and vomiting	Weakness of right limbs	Generalized tonic clonic seizures	Generalized tonic clonic seizures	Headache, nausea and vomiting	Headache and weakness of left limbs	Dizziness and weakness of right foot	Generalized tonic clonic seizures	Weakness of left limbs
Site of lesion	Left temporal lobe	Right frontal lobe	Left parietal lobe and corpus callosum	Bilateral frontal lobe and corpus callosum	Right frontal, temporal, insular lobe and basal ganglia	Right frontal lobe, corona radiata, basal ganglia and corpus callosum	Right frontal and insular lobe	Left frontal and parietal lobe	Left frontal lobe	Right frontal and parietal lobe
Initial OP and adjuvant Tx	GTR + CCRT + TMZ#12	GTR + CCRT + TMZ#2	STR + CCRT	Biopsy + CCRT + TMZ#24 (continuously)	GTR + CCRT + TMZ#22 (continuously)	STR + CCRT	STR + CCRT	STR + CCRT + TMZ/DDP#4 + TMZ#6	GTR + CCRT + TMZ#3	GTR + Dianhydrodulcitol#12
2nd OP and adjuvant Tx	GTR + RT + TMZ#12	NA	Conservative	NA	NA	Conservative	Conservative	Conservative	STR + TG02#1	GTR
H3F3A	G34R	G34R	G34V	G34R	G34R	G34R	G34R	G34R	G34R	G34R
MGMT promoter	Methylated	Methylated	Un-methylated	Methylated	Methylated	Methylated	Methylated	Methylated	Methylated	Un-methylated
IDH	Wildtype	Wildtype	Wildtype	Wildtype	Wildtype	Wildtype	Wildtype	Wildtype	Wildtype	Wildtype
TERT promoter	Wildtype	Wildtype	Wildtype	Wildtype	Wildtype	Wildtype	Wildtype	Wildtype	Wildtype	Wildtype
Morphology	GBM with primitive neuronal component	GBM	GBM	GBM	GBM	GBM	GBM	GBM	GBM	GBM with primitive neuronal component
Current status	Death	Alive	Death	Alive	Alive	Death	Alive	Alive	Death	Alive
OS (months)	75	24	16	21	18	6	16	13	17	20

*CCRT* concurrent chemotherapy and radiation therapy, *DDP* cisplatin, *GBM* glioblastoma, *GTR* gross total resection, *MGMT* O6-methylguanine-DNA methyltransferase, *NA* not applicable, *RT* radiation therapy, *STR* subtotal resection, *TMZ* temozolomide

For initial treatment, gross total resection (GTR) was performed in five patients, whereas four patients received subtotal resection and one patient only received biopsy. For postoperative adjuvant treatment, nine patients received adjuvant concurrent chemotherapy and radiation therapy (CCRT), and one patient who was included in a clinical trial received 12 courses of dianhydrodulcitol. Among the nine patients receiving CCRT, five patients further received maintenance temozolomide (TMZ) treatment and one patient received four courses of TMZ plus cisplatin followed by maintenance TMZ treatment.

Seven patients showed recurrent disease after the initial treatment, and four patients died. Four patients who initially received subtotal resection chose conservative treatment after recurrence, and two died at 6 and 16 months after the initial operation. The remaining three patients with recurrent disease received a second operation, and two of them further received adjuvant therapy after the second operation. Patient 9 received TG02, a novel pyrimidine-based multi-kinase inhibitor of CDKs together with JAK2 and FLT3, after STR for recurrent disease. This patient died at 1 year after recurrence with an OS of 17 months. Patient 1, who was diagnosed with methylated MGMT promoter after initially receiving GTR followed by CCRT and 12 courses of maintenance TMZ treatment, then again underwent GTR followed by radiotherapy and 12 courses of maintenance TMZ treatment for her recurrent disease; this patient achieved the longest OS of 75 months. Moreover, three patients who were also diagnosed with methylated MGMT promoter after initially receiving GTR followed by CCRT and maintenance TMZ treatment were free of recurrence after at least 18 months since the initial operation. Intriguingly, two of the three patients without recurrent disease continuously received maintenance TMZ treatment (ongoing), including patient 4, who initially received only biopsy. These results indicated that only GTR and long-term maintenance TMZ treatment might benefit patients with G34-DHG in conventional treatment.

### Pathological characteristics of G34-DHGs

**Fig. 1:**
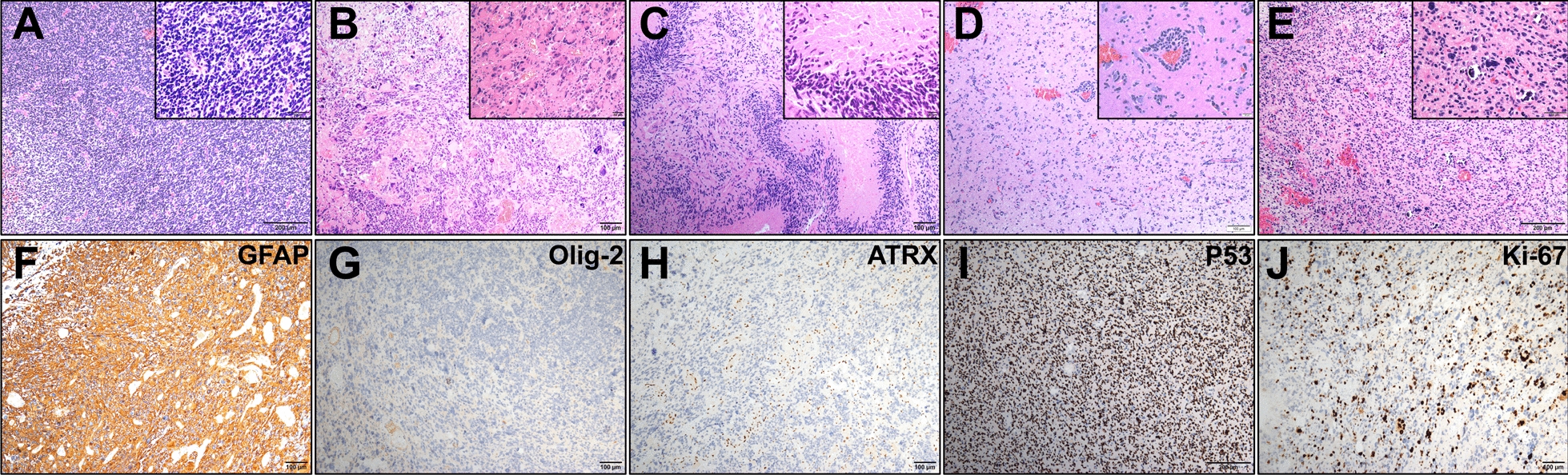
Histological analysis of diffuse hemispheric glioma H3 G34-mutant specimens. Hematoxylin and eosin staining shows embryonal appearance/small cell glioblastoma-like morphology (**A**), giant cell glioblastoma-like morphology (**B**), palisade necrosis (**C**), perivascular growth pattern (**D**) and calcification (**E**). **F**–**J** Immunohistochemistry for the indicated proteins

Histological features including necrosis, nuclear atypia, mitotic activity and vascular characteristics were assessed. Histopathologic examination of the surgical specimens from our cohort showed high-grade glioma morphology (Fig. 1). All cases showed high cell density, featuring nuclear atypia, mitotic activity and cellular pleomorphism with focal gemistocytic cells and multinuclear giant cells. Most cases were glioblastoma (GBM)-like with microvascular proliferating and/or palisade necrosis and two cases showed focal embryonal appearance. However, calcification, perivascular growth pattern and perineuronal satellitosis, which rarely appears in GBM, were also observed (Fig. 1E, F). All cases showed GFAP expression and negative expression of OLIG2; most cases were negative for ATRX (9/10) and most showed diffuse strong p53 positivity (8/10). The Ki67 labeling index was high, ranging from 20 to 60%. Sanger sequencing revealed that all cases were *IDH* wild-type and *TERT* promoter wild-type (Table 1). Methylation PCR revealed that most cases showed MGMT promoter methylation (8/10) (Table 1).

### Mutational landscape of G34-DHGs

**Fig. 2:**
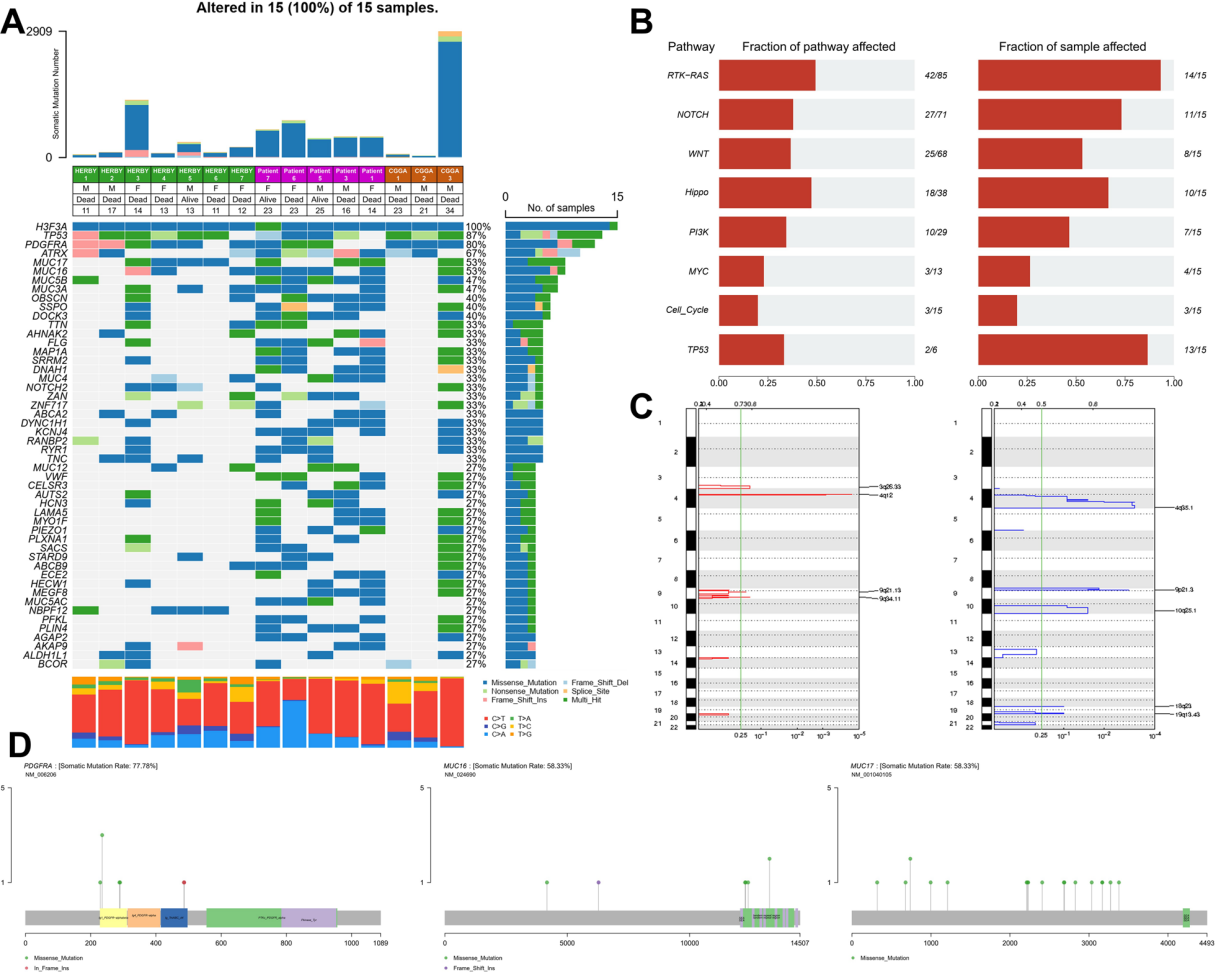
Mutation spectrum of diffuse hemispheric glioma H3 G34-mutant tumors. **A** The total somatic mutations of 15 diffuse hemispheric glioma H3 G34-mutant specimens. Red represents C > T/G > A mutations, blue represents C > G/G > C mutations, green represents T > C/A > G mutations, purple represents C > A/G > T mutations, orange represents T > G/A > C mutations, and yellow represents T > A/ A > T mutations. The percentages indicate the proportion of samples with the mutations. **B** The pathways most commonly affected by genetic mutations in diffuse hemispheric glioma H3 G34-mutant tumors included RTK-RAS, NOTCH, WNT, Hippo, PI3K, TP53, MYC and Cell_Cycle pathways. Left: histogram shows the number of mutations in each pathway. Right: histogram represent the fraction of samples affected. **C** Recurrent copy number alterations. GISTIC2.0 plot of recurrent focal losses (**a**) and gains (**b**). Chromosomes are represented along the vertical axis; q values are marked along the horizontal axis. The green lines mark the cut-off for the significance threshold (q = 0.25). **D** Schematics showing the locations of the missense mutations and truncating mutations on the PDGFRA (left), MUC16 (middle) and MUC17 (right) genes

To investigate the mutational landscape of G34-DHGs, we performed WES on five fresh samples and matched peripheral blood leucocytes from our cohort. The average depths of targeted exome regions in tumors and matched blood samples were 400× and 100× , respectively. More than 98.69% of the targeted regions were covered sufficiently for confident variant calling (≥ 10× depth). We included three G34-DHG cases from the CGGA and seven G34-DHG cases from the HERBY trial (study BO25041; clinicaltrials.gov NCT01390948). The total somatic mutations of the 15 G34-DHG samples are listed in Fig. 2.

The mutational frequencies, mutation types and clinical features of the 15 G34-DHGs are displayed in Fig. 2A. *TP53* (13/15 87.0%), *PDGFRA* (12/15, 80.0%) and *ATRX* (10/15, 67.0%) were the most three frequently mutated genes. Other frequently mutated genes including *MUC17* (8/15, 53.0%), *MUC16* (8/15, 53.0%), *MUC5B* (7/15, 47.0%), *MUC3A* (7/15, 47.0%), *OBSCN* (6/15, 40.0%), *SSPO* (6/15, 40.0%) and *DOCK3* (6/15, 40.0%) were identified by WES.

Notably, MUC family gene mutations have not been reported in G34-DHGs. The human MUC16 gene is located on chromosome 19p13.2 and the MUC17 gene is located on chromosome 7q22.1. Out of the eight cases with *MUC16* mutations, 87.5% (7/8) of the cases had missense mutations, and the remaining case had a frame_shift_ins mutation. Moreover, all eight cases with *MUC17* mutations had missense mutations.

#### Somatic SNVs and indels

A total of 8285 exonic mutations were identified in the 15 G34-DHG patients. Of these mutations, 7187 were missense mutations, 448 were nonsense mutations, 153 were frameshift deletions, 313 were frameshift insertions, 10 were in_frame_del mutations, 1 was an in_frame_ins mutation and 173 were splice site mutations. We removed 1561 silent variants with unknown function. The predominant types of nucleotide substitutions in SNVs in G34-DHGs were C > T/G > A transitions and C > A/G > T transversions.

#### Oncogenic pathways analysis

We identified multiple pathways of somatic mutated genes in G34-DHGs using the oncogenic pathways module of the R package maftools. Crucial signal transduction pathways included the RTK-RAS, NOTCH, WNT, Hippo, PI3K, TP53, MYC and Cell_Cycle pathways (Fig. 2B). Exome sequencing revealed that 14 of 15 cases had changes in the receptor tyrosine kinase RTK-RAS pathway, involving 42/84 pathway genes (including *PDGFRA*, *MET*, *BRAF*, *ERF*, *FGFR1* and *NF1*). KEGG pathway analysis revealed that many of the mutated genes were involved in cancer signal transduction. Mutant genes may promote tumor cell proliferation and escape apoptosis through cascade reaction.

#### Copy number alterations (CNAs)

We conducted somatic CNA analyses in the five SYSUCC cases and seven HEBRY cases. The results identified recurrent gains in chromosomes 3p26.33 (11/12), 4p12 (11/12), 9q21.13 (7/12) and 9q34.11 (4/12). Recurrent losses were identified in chromosomal regions 4p35.1 (10/12), 10q25.1 (10/12), 19p13.43 (9/12), 18q23 (8/12) and 9p21.3 (7/12). Loss of somatic CNAs on chromosome 10q25 affects the largest number of genes (1330 genes), including the *MGMT* locus. Gains of somatic CNAs on chromosome 3p26 affects 396 genes, including *ABCC5*. Studies have shown that ABCC5 is associated with chemoresistance of astrocytic tumors [6].

### PDGFRA mutation and G34-DHGs

PDGFRA is an important receptor tyrosine kinase in glial development and a recurrent driver in high-grade gliomas [7–9] *PDGFRA* mutation and the PDGFRA signaling pathway were reported to play potent oncogenic roles in G34-DHGs [10]. Our sequencing results also showed that most G34-DHGs had *PDGFRA* mutation (12/15).

**Fig. 3:**
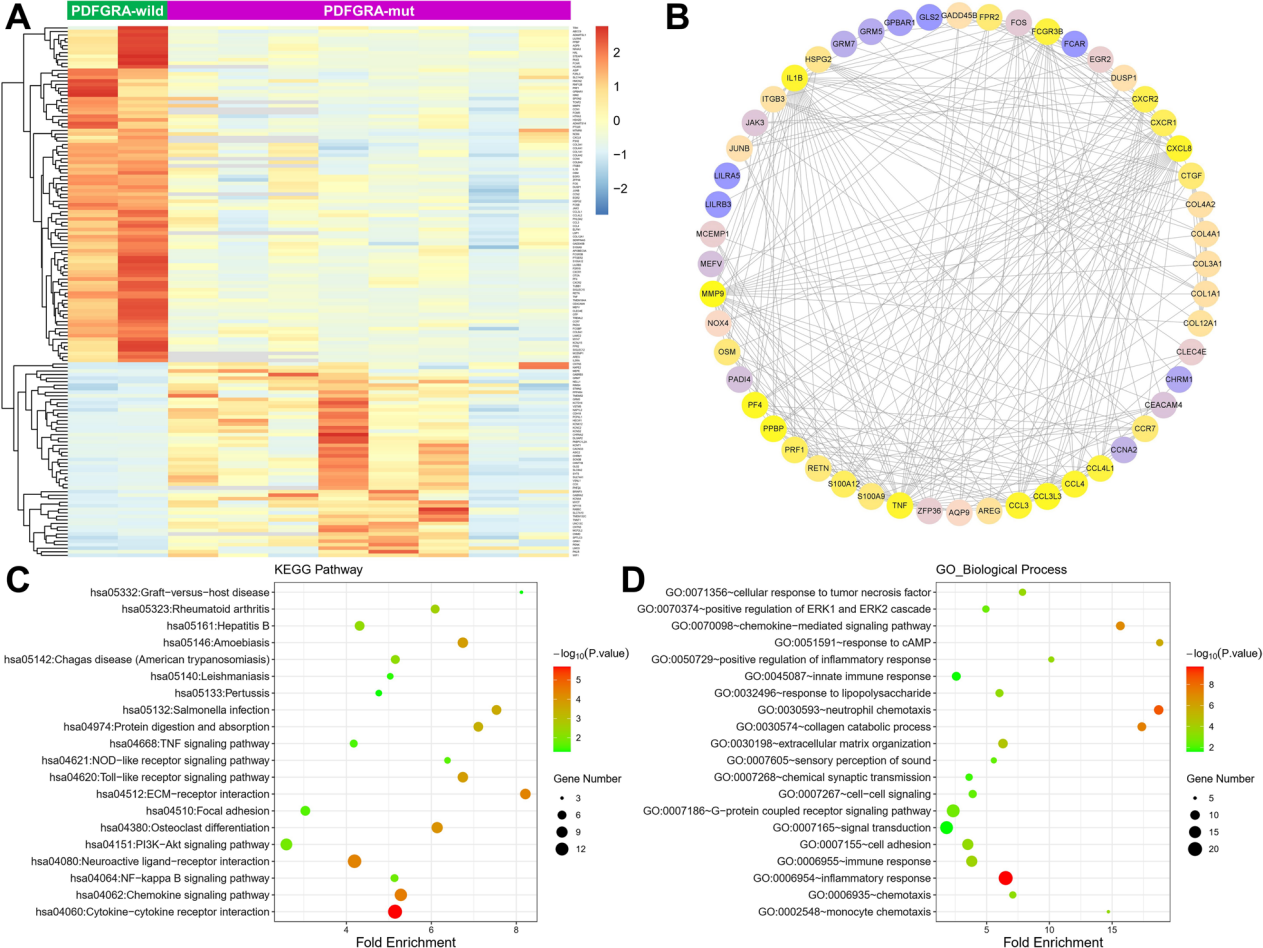
Analysis of differentially expressed genes in diffuse hemispheric glioma H3 G34-mutant tumors with *PDGFRA* mutation. Differential gene expression in two G34-DHG patients with wild-type *PDGFRA* and eight G34-DHG patients with mutated *PDGFRA* (**A**). Protein–protein interactions of the differentially expressed genes (**B**), KEGG pathway analysis (**C**) and GO-Biological process analysis (**D**)

To explore the potential pathways and genes related to *PDGFRA* mutation in G34-DHGs, we analyzed differentially expressed genes (DEGs) using RNA-Seq data from two G34-DHG patients with wild-type *PDGFRA* and eight G34-DHG patients with mutated *PDGFRA*. The results identified 150 DEGs (|FC|≥ 2.0 and P < 0.05), including 95 down-regulated genes and 55 up-regulated genes. The DEGs between the two groups are shown in a heatmap in Fig. 3A. We identified the top 10 hub genes ranked by degree, including *FOS*, *CXCL8*, *CXCR1*, *IL1B*, *COL1A1*, *MMP9*, *FCGR3B*, *TNF*, *CCL4* and *CCL3*. We used MCODE in Cytoscape to identify gene modules in the PPI network and mapped the interaction network of 52 core genes in module 1 (Fig. 3B). The results indicated that the genes were mainly involved with blood microparticles, the phospholipase C-activating G protein-coupled receptor signaling pathway, substrate-specific channel activity, complement and coagulation cascades, the neuroactive ligand-receptor interaction and aldosterone-regulated sodium reabsorption.

We used the DAVID database to analyze the GO and KEGG pathways of the DEGs. KEGG pathway analysis revealed that DEGs were significantly enriched in the extracellular matrix–receptor interaction, cytokine-cytokine receptor interaction, PIK3-AKT signaling pathway and chemokine signaling pathway (Fig. 3C). The enriched GO-Biological Process terms included immune and inflammatory response (Fig. 3D).

### *MUC16* mutation and immune infiltration characteristics of G34-DHGs

**Fig. 4:**
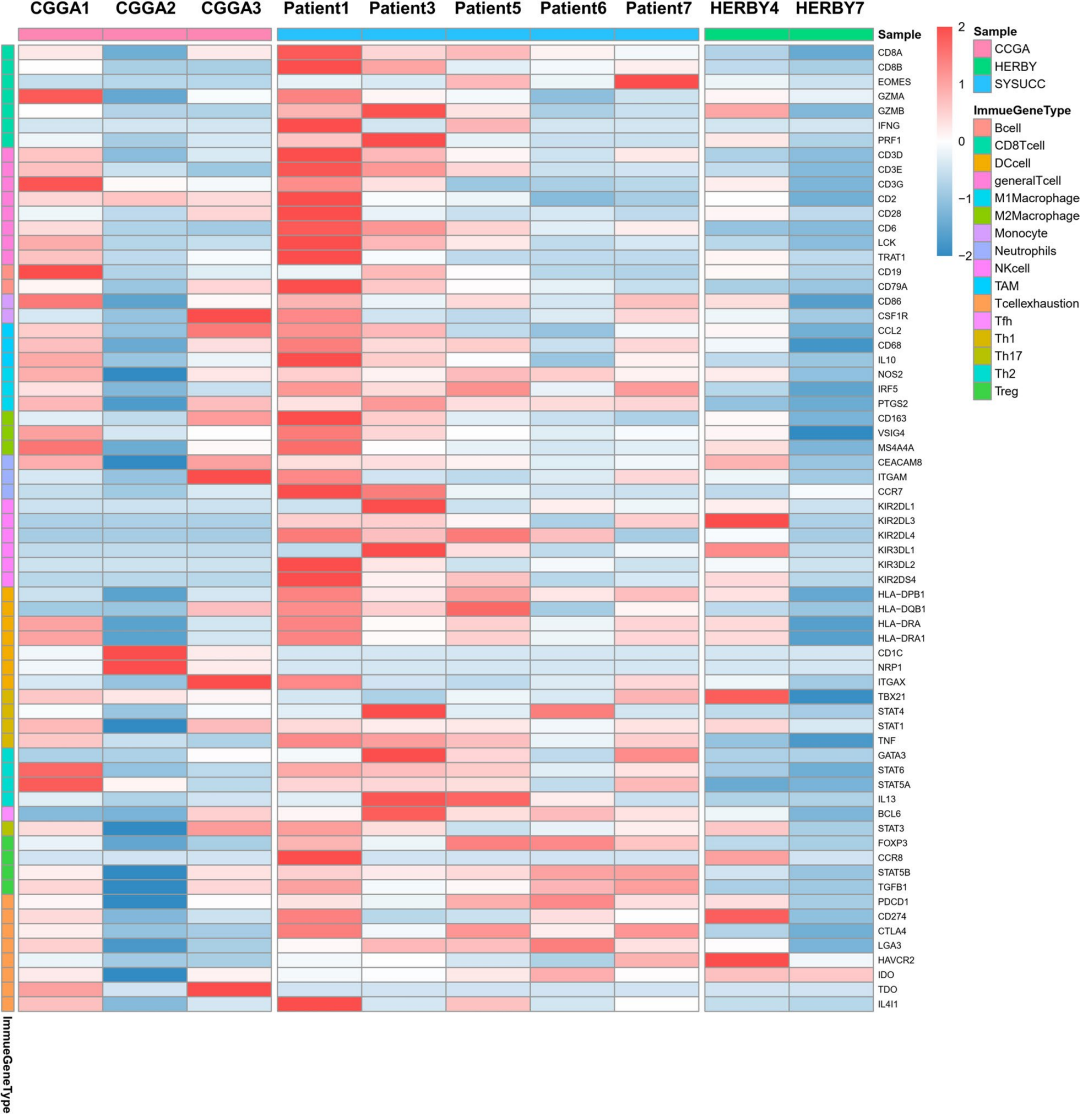
Immune-related genes expression profiles for immune cells plotted as a heatmap from 10 cases with RNA-seq data

Previous studies indicated that pHGGs with a high mutation load have an elevated neoantigen load and immune response [11, 12]. However, tumors with histone H3 gene mutations are considered as immune cold tumors, which are defined as a lack of CD8 immunoreactivity and lack of tumor-infiltrating lymphocytes [13]. Using the RNA sequencing results, we next analyzed the expressions of immune-related genes in 10 patients with G34-DHGs, including 5 from SYSUCC, 3 from CGGA and 2 of the HERBY cases. The 67 differentially expressed immune-related genes were classified according to CD8 + T cell, T cell (general), B cells, monocyte, tumor-associated macrophage, M1 macrophage, M2 macrophage, neutrophil, natural killer cell, dendritic cell, Th1, Th2, Tfh, Th17, Treg and T cell exhaustion markers (Fig. 4). In general, consistent with HERBY Phase II Randomized Trial, we also found G34-DHGs were immune cold tumors, with a few exceptions. For instance, the tumor specimen of patient 1 in our cohort showed significant immune infiltration with substantial amounts of CD4 and CD8 T cells (Additional file 2: Figure S2). The OS of this patient reached 75 months, which was markedly longer than that of the nine patients with low immune infiltration (mean survival time: 12 months).

A previously published pan-cancer analysis of 30 solid tumor types showed that patients with *MUC16* mutations showed higher tumor mutation burden and neoantigen burden, indicating increased tumor immunogenicity, which can predict immune checkpoint inhibitor treatment response. The study included 397 cases of glioblastoma and 61 of these cases (15.37%) showed *MUC16* mutations [14]. Therefore, we wondered whether the *MUC16* mutation in G34-DHGs might also be associated with immune infiltration.

**Fig. 5:**
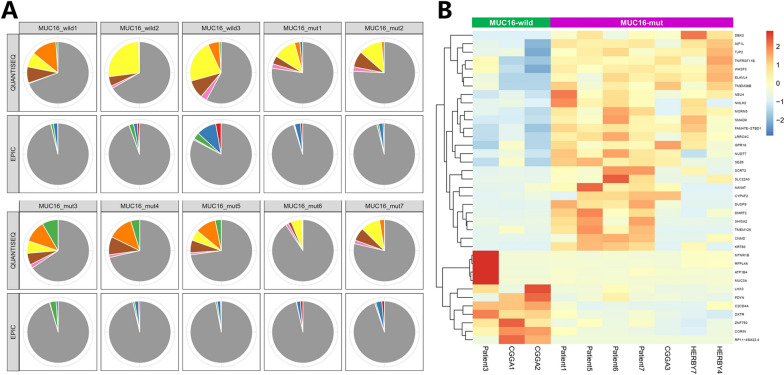
Immune gene expression signatures and differential genes for *MUC16* mutant and wild-type diffuse hemispheric glioma H3 G34-mutant tumors. **A** A multi-panel pie plot showing the proportion of immune cell types in *MUC16* mutant and wild-type samples. The eight immune cell types are highlighted in different colors. **B** Differential gene expression types in *MUC16* mutant and wild-type cases

We used TIMER 2.0 to analyze the immune cell infiltration of G34-DHGs with *MUC16* mutation and G34-DHGs with wild-type *MUC16*. However, no connection between *MUC16* mutation and immune cell infiltration was found; this may be because of the small number of cases and relatively low immune infiltration of G34 glioma. We further analyzed the DEGs between G34-DHGs with *MUC16* mutation and G34-DHGs with wild-type *MUC16*. G Protein-Coupled Receptors (GPCR) signaling relevant proteins MTNR1B, OXTR and PDYN were all low expressed in G34-DHGs with *MUC16* mutation (Fig. 5).

### Survival analysis of patients with G34-DHGs

**Fig. 6:**
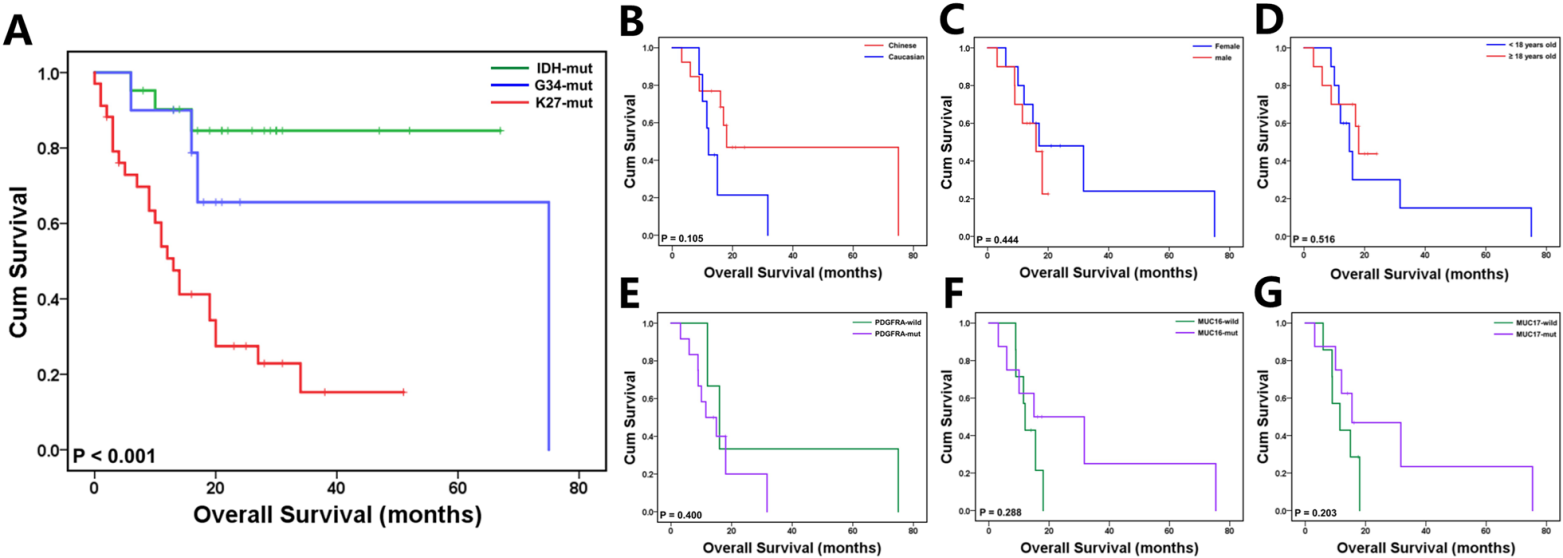
Kaplan–Meier curves of overall survival (OS). **A** Overall survival analysis of patients with G34-DHGs compared with patients with *IDH*-mutant HGGs and patients with H3K27M DMGs in our cohort (SYSUCC); **B** overall survival of Chinese (SYSUCC + CGGA) and Caucasian (HERBY Trail) patients with diffuse hemispheric glioma H3 G34-mutant tumors; **C** overall survival of male patients and female patients in the overall patient group (SYSUCC + CGGA + HERBY Trail); **D** overall survival according to age (age ≥ 18 years vs. < 18 years) in the overall patient group (SYSUCC + CGGA + HERBY Trail); **E** overall survival according to *PDGFRA* mutation (*PDGFRA* mutation vs. *PDGFRA* wild-type) in G34 WES cases; **F** overall survival according to *MUC16* mutation (*MUC16* mutated vs. *MUC16* wild-type) in G34 WES cases; **G** overall survival according to *MUC17* mutation (*MUC17* mutated vs. *MUC17* wild-type) in G34 WES cases

We performed survival analyses of the patients from our center (SYSUCC) (Fig. 6). We found that the OS of patients with H3G34-mutant DHGs was worse than that of patients with IDH-mutant high-grade gliomas, but better than that of patients with H3K27M-mutant DMGs. The mean survival times of patients with IDH-mutant high-grade gliomas, G34-DHGs and H3K27M DMGs were 58.4, 53.8 and 18.4 months, respectively (P < 0.001).

We further performed analysis in the overall patient group (patients with G34-DHGs from SYSUCC, CGGA and HERBY Trial). Age (≥ 18 years old vs. < 18 years old) and sex (male vs. female) did not influence patient prognosis. Although no statistical difference was achieved, Kaplan–Meier curve analyses showed some trends according to race, *PDGFRA* mutation, *MUC16* mutation and *MUC17* mutation. The median OS for Chinese patients was 18 months compared with 12 months for Caucasian patients (P = 0.105). Patients with *PDGFRA* mutation tended to show a shorter OS (median OS: 11.5 months vs. 16 months), and *MUC16* and *MUC17* mutations both seemed favorable for prognosis (median OS: 15 months vs. 12 months and 16 months vs. 11.5 months, respectively). Importantly, although immune infiltration varied in G34 glioma patients, patient 1 in our cohort who harbored MUC16 mutation had obviously high immune infiltration and achieved the longest OS of 75 months.

### External validation

To validate that PDGFRA and MUC family gene mutations status in G34-DHGs, we reviewed the reports of tumor-sequencing test from 6 external patients, among which WES, a big panel targeting 539 or a small panel targeting 135 tumor genes were respectively performed in every 2 patients (Table 2). Somatic alterations in PDGFRA were detected in all 6 external patients, including 2 patients having both missense mutations and gene amplification, 1 patient having a missense mutation only, 1 patient having both a missense mutation and a deletion-insertion variant, and 2 patients having in-frame insertions or deletions. For the 2 patients who received WES, missense mutations in MUC4 and MUC12 were detected individually. Unfortunately, the targeted sequencing panels did not include MUC family genes in the panel.

**Table 2:** Somatic alterations in PDGFRA and MUC family in G34-DHGs of 6 external patients

Patients	Sex/age (years)	Sequencing test	Somatic alterations in *PDGFRA*	Somatic alterations in *MUC* family	Other important molecular changes
ExP1	M/22	WES	p.E387del	MUC4 p.V1961L	ATRX p.K1584Ifs, EGFR amplification
ExP2	F/18	WES	p.L275S Amplification	MUC12 p.T1733A	ATRX p.Q2168Rfs, TP53 p.R306&p.R342
ExP3	F/37	539 genes panel	p.E279_A280delinsT p.Y288C	N/A	TP53 p.W53&p.R342 CDKN2A deletion
ExP4	M/10	539 genes panel	p.C290G Amplification	N/A	ATRX c.5955_5956 + 2del TP53 p.E171del
ExP5	M/21	135 genes panel	p.Q246_Y249delinsH	N/A	ATRX p.R781 TP53 p.V147Lfs
ExP6	M/32	135 genes panel	p.G286E	N/A	ATRX c.5567 TP53 c.524

## Discussion

Diffuse hemispheric glioma H3 G34-mutant is a newly recognized tumor type in the 2021 WHO Classification of Tumors of the Central Nervous System. This tumor is a malignant infiltrative glioma that typically occurs in the cerebral hemispheres and harbors a missense mutation in the *H3F3A* gene that results in a G34R/V substitution in histone H3. The histological heterogeneity of G34-DHGs has been previously reported [15]. In general, most cases show high-grade anaplastic features, with microscopic features of anaplastic astrocytoma, GBM or embryonic tumors (primitive neuroectodermal tumor-like features) or even anaplastic pleomorphic xanthoastrocytoma [16]. Therefore, misdiagnosis of cases is likely if clinicians and pathologists rely solely on histological diagnosis. Consistent with these findings, most cases in our center also presented as GBM-like and with/without a focal embryonal appearance. However, calcification, perivascular growth pattern and perineuronal satellitosis, which rarely appear in GBM or called ‘secondary structures’, were observed [3, 17]. We further analyzed the immunohistochemical and characteristics of H3G34-mutant diffuse gliomas. Lack of OLIG2 expression is one of the characteristics of this tumor and was previously reported in 100% of G34 DHGs [18]. We also observed loss of OLIG2 expression in all of our cases. In addition, loss of ATRX expression and p53 overexpression are also frequently observed in this tumor type. The *H3F3A* G34R/V mutation is associated with a high frequency of MGMT methylation, but this is mutually exclusive with IDH or TERT promoter mutation.

Significantly, we found frequent *PDGFRA* mutation (12/15) in the G34-DHGs in our study. PDGFRA is a classical receptor tyrosine kinase with five extracellular immunoglobulin-like structures and responsive to platelet-derived growth factor (PDGF) [19], and this protein plays an important role in cell determination, cell proliferation and migration during neural development and adult neurogenesis [19, 20]. In glioma, somatic alterations of PDGFRA include missense mutations, in-frame insertions or deletions, gene amplification and fusion. A high proportion of gliomas, especially TERTp wild-type GBM, are associated with somatic alterations in *PDGFRA*. Recurrent *PDGFRA* mutations have also been found in low-grade neurotumors associated with refractory seizures, called septal dysplasia neuroepithelial tumors or mucinous glial neuronal tumors [21]. *PDGFRA* gene amplification is present in approximately one-third of HGGs, and most tumors with *PDGFRA* gene amplification have genomic deletions of exons 8 and 9[22]. Ozawa et al. reported an adult case with a gene fusion between *PDGFRA* and *VEGFR2* [23]. Point mutations are located in the extracellular domain, transmembrane domain and kinase domain of the PDGFRA receptor. These oncogenic mutations lead to constitutively activated PDGFRα, thereby activating downstream signal transduction [24]. Aberrant PDGFRA signaling in gliomas leads to activation of the Ras-Raf-MEK-ERK pathway [25]. Gain-of-function PDGFRA variants trigger multiple tumor-promoting signaling cascades, including phospholipase Cγ (PLCγ), phosphatidylinositol-3-kinase (PI3K), mitogen-activated protein (MAP) kinase, and signal transduction and Activators of Transcription (STATs) [26, 27]. Targeting PDGFRA by tyrosine kinase inhibitors (TKIs) or antibodies showed promising antitumor effects in patients with various PDGFR-driven extracranial tumors and pre-clinical models of gliomas [28–31]. In a glioma subset with histone H3 mutation (H3K27M DMGs), *PDGFRA* amplification and mutation were reported together with histone H3.3 mutation. Li et al. found that 18 cases of 112 midline glioma patients had *PDGFRA* mutations [32]. Both our study and Chen’s study found high frequencies of *PDGFRA* mutations in G34-DHGs [10]. *PDGFRA* mutation was present in 12 out of 15 cases (80%) in our study group, and Chen et al. extensively analyzed 95 cases of HGGs with H3.3G34R/V mutation and found that *PDGFRA* mutations presented in 44% of all tumors and 81% of recurrent tumors, they speculated that *PDGFRA* mutant G34 tumors have expanded astrocytic compartments that are conducive to maintaining the active chromatin conformation state of the GSX2 enhancer to maintain high PDGFRA expression and continue to promote the carcinogenic state [10]. In GBM and in *IDH* mutant lower-grade (WHO Grades II/III) glioma, *PDGFRA* amplification is associated with shorter progression-free survival and OS and it is an independent prognostic factor [33]. *PDGFRA* amplification was also an indicator of poor prognosis in H3K27M DMGs [34, 35]. However, the prognostic implication of *PDGFRA* mutation has not been extensively studied. Our data suggested that PDGFRA mutation may be an indicator for poor prognosis in G34-DHGs, but the results were not statistically significant; this may because of the relatively small number of G34 cases in our cohort.

Another important finding included the MUC family genes were also frequently mutated in G34-DHGs. The MUC gene family encodes mucins, a type of high molecular weight glycoprotein [36]. There are 21 MUC genes in the human genome that encode secreted and membrane-type mucins [37]. Increasing evidence has shown that MUC proteins play an important role in regulating tumor cell proliferation, growth, apoptosis and chemical tolerance [38, 39]. MUC gene mutation analysis showed that *MUC16* (OMIM 606154) has the highest mutation frequency in G34-DHGs, followed by *MUC17*. A pan-cancer analysis involving somatic mutations of 10,195 samples and mRNA expression profiles of 9850 samples for 30 solid tumors found a greater abundance of immune cells in the microenvironment of *MUC16*-mutated tumors, and *MUC16* mutation was associated with factors associated with response to immune checkpoint inhibitor therapy[14]. Tumors with *MUC16* mutations exhibited a higher tumor mutational burden and more abundant neoantigen compared with that of *MUC16* wild-type tumors, indicating increased tumor immunogenicity. *MUC16*-mutated tumors were characterized by upregulated expression of T-effector and interferon-γ gene signature, a hallmark of preexisting immunity associated with pronounced benefit from checkpoint blockade. An additional hallmark of *MUC16*-mutated tumors is the augmented expression of multiple inhibitory checkpoints including LAG3 and others, suggesting potential adaptive immune resistance to anti-PD-1/PD-L1 therapies and that additional inhibitory pathways beyond the PD-1/PD-L1 axis might be targets. Although we discovered a high frequency of *MUC16* mutation in G34-DHGs, only one case in the *MUC16* mutation group showed abnormal high immune infiltration with abundant tumor-infiltrating lymphocytes and this patient survived up to 75 months. We did not find a relationship between *MUC16* and immune infiltration in G34-DHGs, which may be from the small sample numbers and immune-cold characteristics of G34-DHGs.

Regarding survival analysis, one study reported that the survival of patients with G34-DHGs was as poor as survival of patients with K27M DMGs [13]. However, all the cases were children in the study and the sample size was small, which may have resulted in bias. Our conclusion is consistent with most studies, showing that the OS of patients with H3 G34-mutant DHGs was worse than the survival of patients with IDH-mutant GBMs, but better than the survival of patients with H3 K27M-mutant DMGs; this may be partly because of the high frequency of MGMT promoter methylation in these tumors and favorable response to TMZ chemotherapy [8].

It is clear that histone mutations represent clearly defined entities within an umbrella HGG classification, and will require therapeutic development and clinical trials distinct. Compared with H3K27M DMGs, G34-DHGs were not sensitive to ONC-201, an effective therapeutic drug for H3K27M-DMGs [40]. Most G34-DHG patients had *MGMT* promoter methylation, and the rate was higher than the low incidence rate in H3K27M-DMGs (only one case had MGMT promoter methylation in our cohort, 1/34; data not shown), indicating that GTR and long-term maintenance TMZ treatment might benefit patients with G34-DHGs. Most cases also had *PDGFRA* mutations, and therefore the PDGFRA signaling pathway could be a potential therapeutic target. In addition, immunotherapy may be an option for cases with substantial immune infiltration and *MUC16* mutation. These important findings will lay the foundation for the future personalized treatment of G34-DHGs, thereby helping to improve the current scenario in which existing treatment methods have not significantly improved the median survival of these patients.

Our study has some limitations. First, the overall sample size was small, mainly because G34 DHG is a brand-new type of pediatric-type diffuse high-grade gliomas in the latest 2021 WHO classification of tumors of the central nervous system, thus, total number of confirmed cases was small and some G34 DHG cases could be going undiagnosed which lack molecule detection. In addition, the study is mainly descriptive and the significance is possible only for survival analysis of different types of glioma but not yet for the definition of prognostic genetic markers, like PDGFRA and MUC16 and 17 mutations, studies on large sample are needed to confirm the prognostic results of this study. Last, prospective studies such as a follow-up clinical trial is needed to access the effects of the various PDGFRA targeted drugs combined with or without TMZ in G34 DHGs, like phase I study using crenolanib to target PDGFR kinase in children and young adults with newly diagnosed DIPG [41].

## Conclusions

G34-DHG is a new high-grade glioma with high frequency of *PDGFRA* and *MUC* gene family mutations. PDGFRA may serve as a poor prognostic indicator and an effective therapeutic target for G34-DHGs. Moreover, *MUC16* mutation tends to be a favorable prognostic factor and indicates high immune infiltration in certain patients, which may provide a new direction for immunotherapy of G34-DHGs.

## Supplementary Information

**Additional file 1: Figure S1.** Diagram of the study flow. We initially examined 230 glioma cases (0–35 years old) from SYSUCC. 30 cases were excluded due to no enough tissue. *IDH1/2* Sanger sequencing was performed in 200 cases, revealing 21 *IDH* mutated and 179 *IDH* wild-type cases. *H3F3A* Sanger sequencing revealed 34 cases with H3 K27M mutation, 9 cases with H3 G34R mutation and 1 case with H3 G34V mutation. The 10 G34R/V cases were reviewed by hematoxylin and eosin and immunohistochemical staining. Only five cases had sufficient fresh tumor samples for whole-exome and RNA sequencing. This study also included three cases from CGGA database and seven cases from the HERBY Trial analyzed and selected as shown.

**Additional file 2: Figure S2.** Substantial T cell infiltration in patient 1. (A) Hematoxylin and eosin staining and immunohistochemistry for (B) CD4 and (C) CD8.

## Data Availability

Data in the study will be deposited in the Research Data Deposit (RDD, http://www.researchdata.org.cn/) of Sun Yat-sen University Cancer Center (SYSUCC), which will be shared by request from any qualified investigator upon approval by the SYSUCC data request committee. All data generated or analysed during this study are included in this published article. And the data is available from the corresponding author on reasonable request.

## Abbreviations

G34-DHG: Diffuse hemispheric glioma H3 G34-mutant
CGGA: Chinese Glioma Genome Atlas database
HE: Hematoxylin–eosin
IHC: Immunohistochemistry
WES: Whole-exome sequencing
pHGG: Pediatric-type diffuse high-grade glioma
SYSUCC: Sun Yat-sen University Cancer Center
OS: Overall survival
GTR: Gross total resection
CCRT: Concurrent chemotherapy and radiation therapy
TMZ: Temozolomide
GBM: Glioblastoma
