# Supplementary figures and images for: High frequency of PDGFRA and MUC family gene mutations in diffuse hemispheric glioma, H3 G34-mutant: a glimmer of hope?
Source: J Transl Med. 2022 Feb 2;20:64. doi: 10.1186/s12967-022-03258-1 (PMC8812218; doi:10.1186/s12967-022-03258-1)

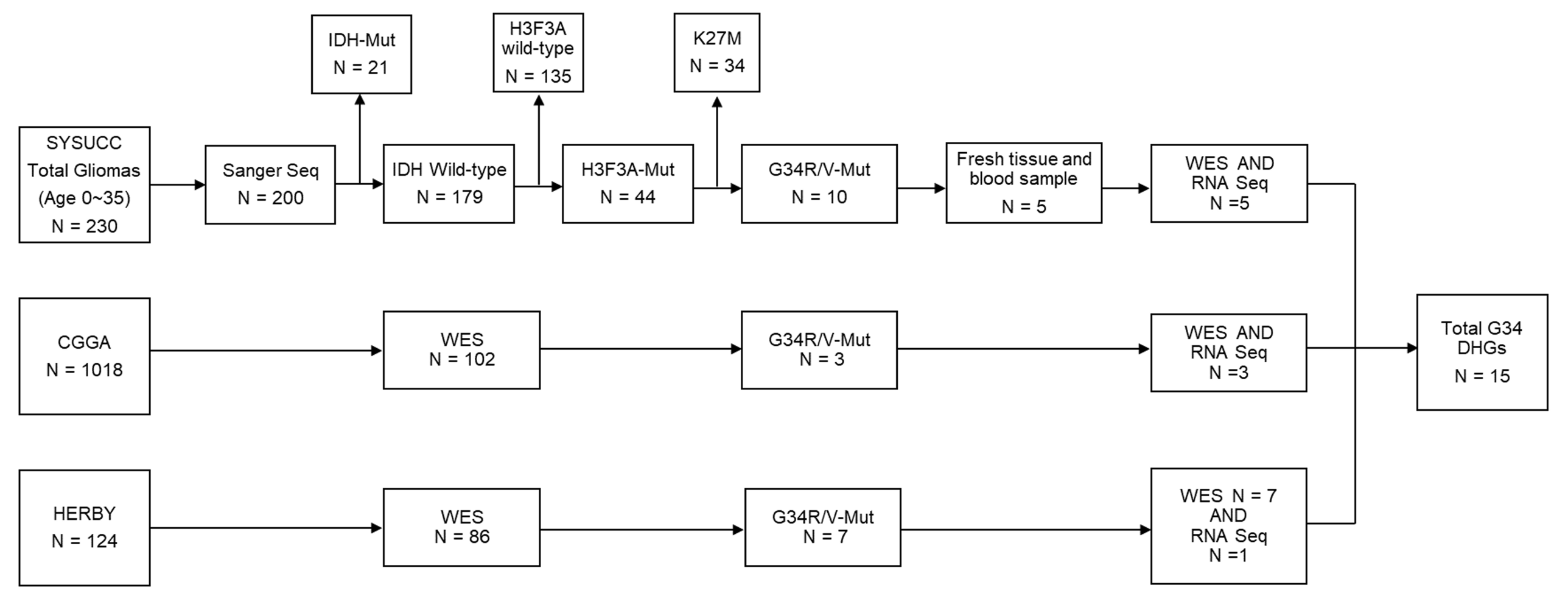

Supplement: Supplementary file 1 — Additional file 1: Figure S1. Diagram of the study flow. We initially examined 230 glioma cases (0–35 years old) from SYSUCC. 30 cases were excluded due to no enough tissue. IDH1/2 Sanger sequencing was performed in 200 cases, revealing 21 IDH mutated and 179 IDH wild-type cases. H3F3A Sanger sequencing revealed 34 cases with H3 K27M mutation, 9 cases with H3 G34R mutation and 1 case with H3 G34V mutation. The 10 G34R/V cases were reviewed by hematoxylin and eosin and immunohistochemical staining. Only five cases had sufficient fresh tumor samples for whole-exome and RNA sequencing. This study also included three cases from CGGA database and seven cases from the HERBY Trial analyzed and selected as shown. [file 12967_2022_3258_MOESM1_ESM.jpg]

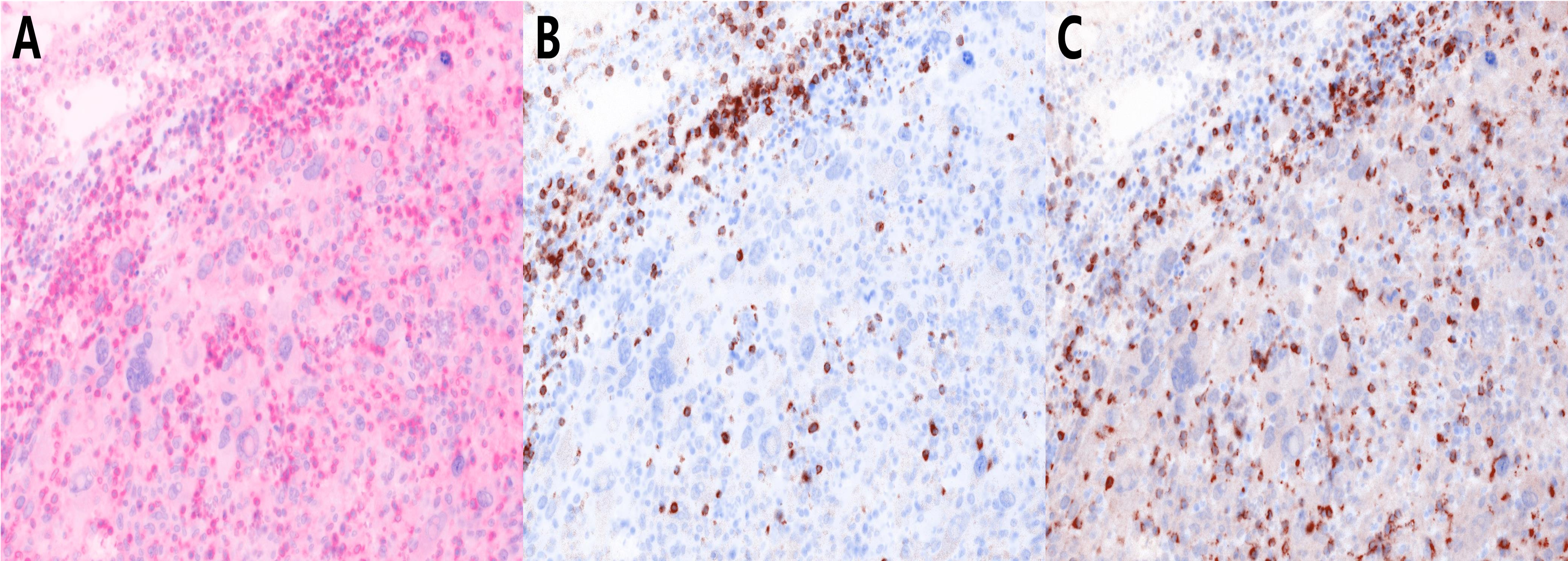

Supplement: Supplementary file 2 — Additional file 2: Figure S2. Substantial T cell infiltration in patient 1. (A) Hematoxylin and eosin staining and immunohistochemistry for (B) CD4 and (C) CD8. [file 12967_2022_3258_MOESM2_ESM.jpg]
